# Visual feedback modulates the 1/*f* structure of movement amplitude time series

**DOI:** 10.1371/journal.pone.0287571

**Published:** 2023-10-20

**Authors:** Andrew B. Slifkin, Jeffrey R. Eder

**Affiliations:** Department of Psychology, Cleveland State University, Cleveland, Ohio, United States of America; The Ohio State University, UNITED STATES

## Abstract

In our prior studies, human participants were required to generate long sequences of targeted hand movement when task difficulty varied between conditions, and where full vision of the hand and target was always available. The movement amplitude—that is, the actual distance travelled—for each movement was measured; consecutive movement amplitude values were formed into time series; then, the time series were submitted to spectral analysis. As task difficulty increased, there was a pink-to-white-noise shift in movement amplitude time-series structure. Those changes could be attributed to a difficulty-induced increase in the need to engage visual feedback processes, which maintain accurate guidance of the hand to the target. The current study was designed to provide a more direct test of the hypothesis that difficulty-induced increases in visual feedback processing modulate movement amplitude time-series structure. To that end, we examined cyclical aiming performance under four unique conditions created from the crossing of two index of difficulty (2 and 5 bits) and two visual feedback (visual feedback and no-visual feedback) conditions. That allowed us to examine how variations in visual feedback quality might influence difficulty-induced changes in time-series structure. In the visual feedback condition, we predicted that the increase in difficulty should result in a pink-to-white-noise shift in time-series structure. If that expected shift resulted from increased engagement of visual feedback processing, then in the no-visual feedback condition—where visual feedback processing was disabled—we should observe a strengthened pink-noise time-series structure that does not change with the increase in difficulty. The current results confirmed those predictions. That provides further support for the hypothesis that engagement of closed-loop visual feedback processing modulates movement amplitude time-series structure.

## Introduction

Traditionally, the control of manual aiming has been understood in terms of two types of information processes: Feedforward and feedback control [e.g., 1–5]. Feedforward control is based on preset, automatic processes with its output expressed through the primary submovement of the movement trajectory. For example, when the target is very wide and there is high tolerance for endpoint variability, movement-related error correction may not be required, and the target may be reached through feedforward control of the primary submovement alone. In contrast, as task difficulty increases, for example, through a reduction in target width, maintenance of endpoint accuracy may require engagement of one or more secondary submovements. The secondary submovements are thought to reflect the operation of closed-loop visual feedback processes that monitor the position of the hand relative to the target and adjust hand position to ensure that it successfully reaches the target.

**Fig 1:**
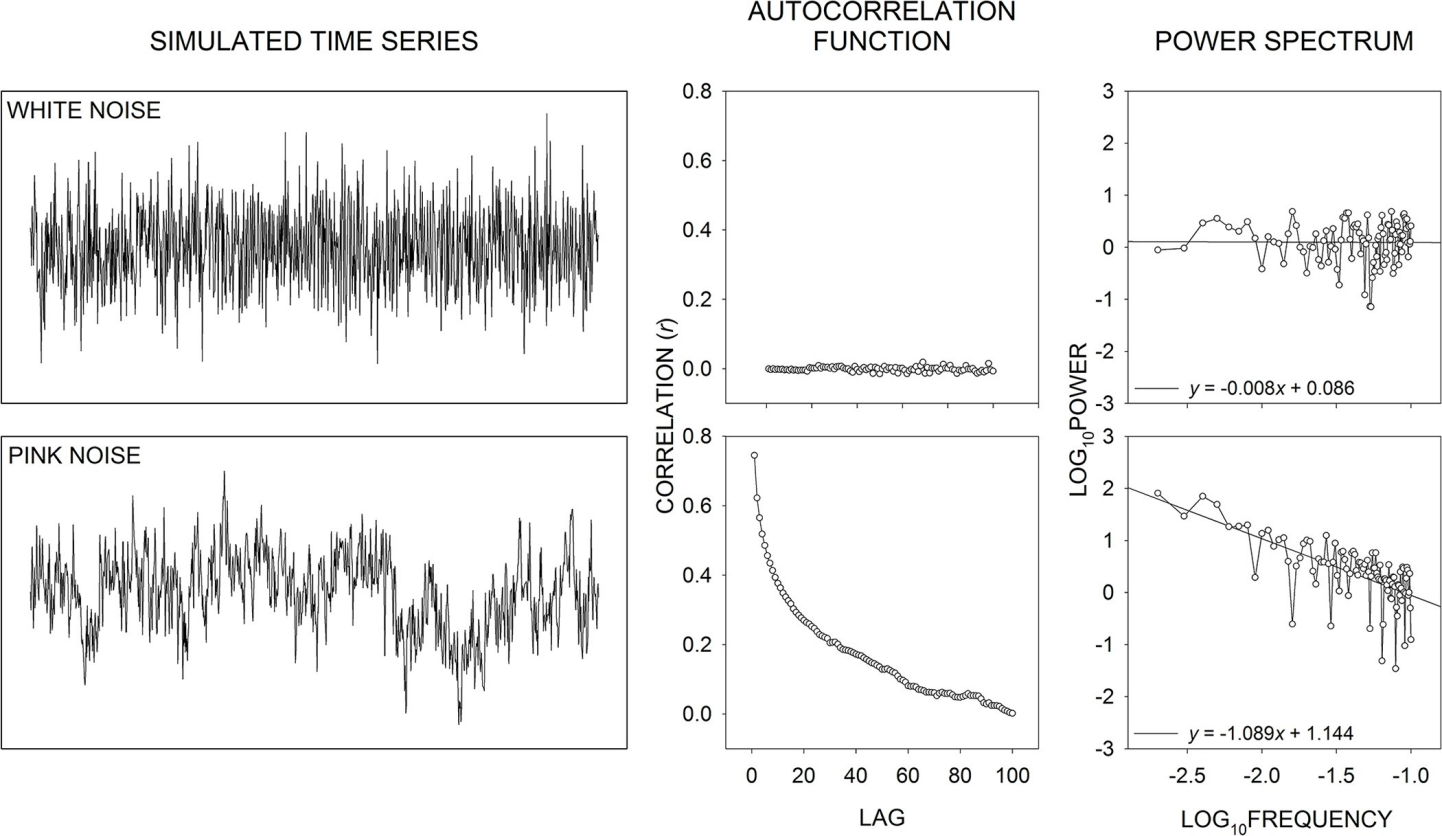
Simulated white-noise and pink-noise time series and associated time-series analyses: The autocorrelation function and the power spectrum. Both the simulated white-noise (top left panel) and pink-noise (bottom left panel) time series contain 1000 events of varying amplitude levels. The autocorrelation reflects the correlation of a time series with itself at different lags. For example, a lag-0 autocorrelation reflects the correlation of each value in the time series with itself. In that case, the correlation will always be *r* = 1. The lag-1 autocorrelation reflects the correlation of each time-series value with the very next time-series value; the lag-100 autocorrelation is the correlation of each time-series value with the value 100 events into the future. In the current figure, the autocorrelation spans lag 1 to lag 100. Another method of time-series analysis is spectral analysis: The power spectrum (right panels) decomposes a signal into component sinusoidal frequencies and assigns a power value scaled to the magnitude of each frequency’s contribution to the time series. For white noise, by definition, each observation in the time series is independent of every other event in the time series, and across all autocorrelation lags the correlations hover around a value of zero (top middle panel). For the white-noise power spectrum, each frequency in the spectrum contributes similar levels of power to the signal; as reflected by the slope of the frequency-power regression equation, there is essentially no change in power as a function of frequency (top right panel). In contrast, for the pink-noise time series one may observe low-frequency wavelike oscillations that reduce in magnitude as frequency increases (bottom left panel). That gives rise to elevated autocorrelations (bottom middle panel) and an inversely proportional reduction in power as a function of frequency: The slope of the frequency-power regression equation is essentially −1 (bottom left panel).

Our research has shown that when participants are required to generate long sequences of cyclical aiming movements under conditions of full vision and movement amplitude—the target-to-target travel distance—is recorded, movement amplitude time-series structure increases in complexity—shifting from pink to white noise—as task difficulty increases [6,7]. Fig 1 provides an illustration of simulated pink and white noise (left panels) along with their associated autocorrelation functions (middle panels) and power spectra (right panels). The autocorrelation reflects the strength of association between the value of each event in a time series and the value of future time-series events. For pink noise, when the distance or time between events (the lag) is short, correlations are high, and as the lag increases, correlations decline (top middle panel). In contrast, for white noise the correlations are maintained at around zero across all lags (bottom middle panel). It is not until about lag 100 that the pink- and white-noise autocorrelation functions converge. Overall, it can be said that pink-noise exhibits positive sequential correlations that can extend into the distant future, whereas white-noise time series exhibit no sequential correlations [e.g., 8, pp. 34–36]. The right panels of Fig 1 show the power spectrum for each time series: It models a time series as a set of sinusoidal functions of different frequencies with amplitude (or power) values assigned to each frequency; each assigned power value reflects the degree to which oscillations at a given frequency contribute to the time series. For pink noise, as frequency increases there is an inversely proportional fall-off of power, whereas for white noise power is constant across the range of frequencies (see Fig 1, right panels). In our past research, as in the current study, time-series structure was quantified through spectral analysis of movement amplitude time series (see “Materials and methods” for more detail): Again, our previous research examined performance under conditions of full vision, and we found that movement amplitude time-series structure shifted from pink to white noise as task difficulty increased [6,7].

**Fig 2:**
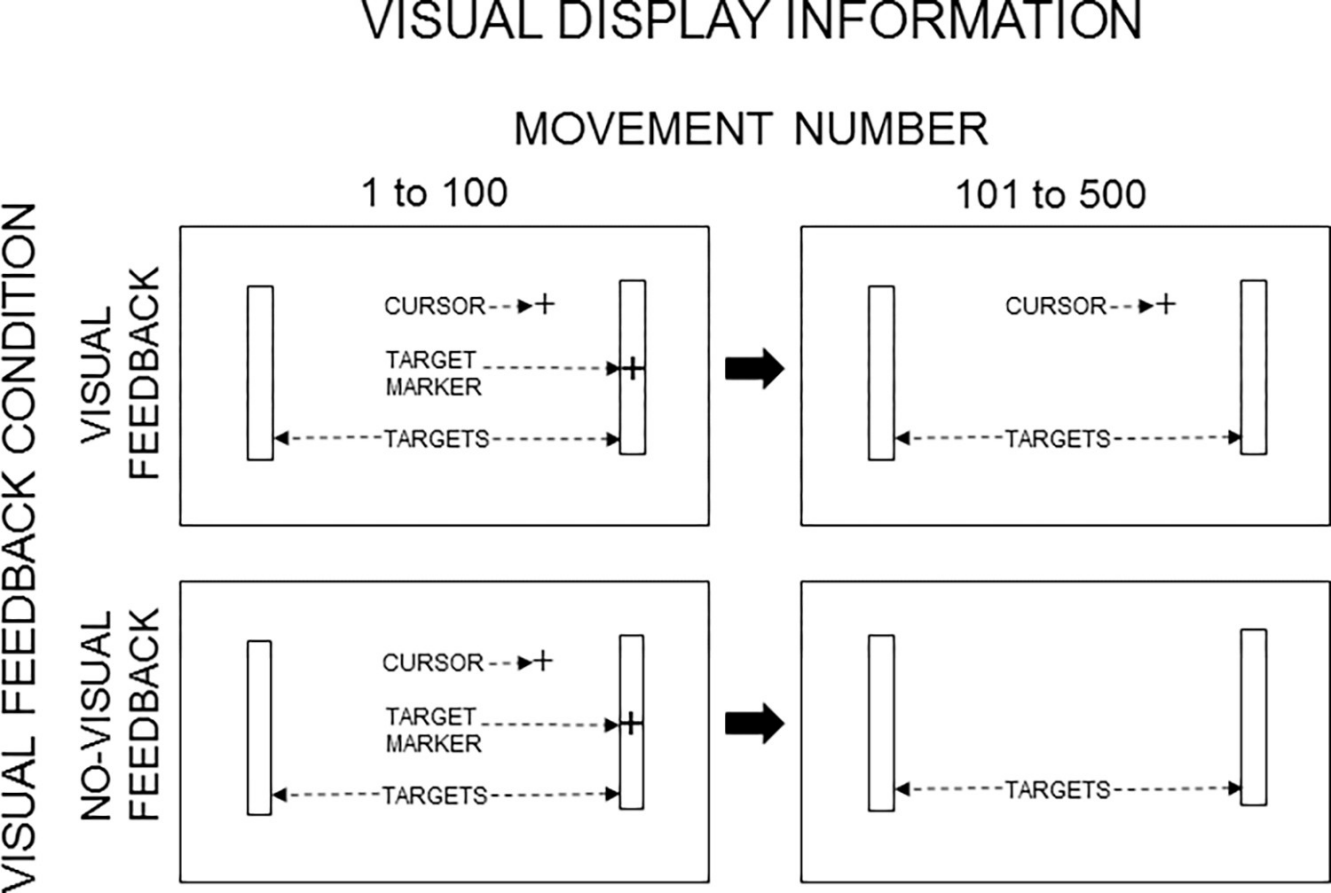
Schematic depiction of visual information displayed in the visual feedback and no-visual feedback conditions. As shown in the left panels, all conditions contained an initial period, over movements 1 to 100, where the targets, a target marker (+) (marking the currently active target), and a mouse-driven cursor (+) were displayed on the video monitor. In addition, the computer sounded an error “beep” (not referenced in the figure) whenever a movement terminated outside the target boundaries. Then, from movements 101 to 500, in the visual feedback condition the targets and cursor remained visible (top right panel), but in the no-visual feedback condition only the targets remained visible (bottom right panel). (Furthermore, during movements 101 to 500, the error “beep” was disabled in all conditions.) Note that the elements in the current illustration have not been drawn to scale; the actual targets appeared as white rectangular outlines superimposed on a black video-screen background; both the target markers and cursor were identical white crosshairs.

In addition, in that research [6,7] we defined task difficulty according to the equation ID = log_2_(2*A*/*W*), where ID is the index of difficulty expressed in binary units (bits), *A* is the amplitude or distance between target centers, and *W* is the target width [9]. We hypothesized that maintenance of aiming accuracy at higher IDs required the engagement of secondary submovements that adjust the amplitude of individual movement trajectories based on closed-loop visual feedback processes: Primary submovement amplitudes that were too short or too long were spatially extended or truncated, respectively, through corrective secondary submovements [e.g., 1,3–5,10–13]. Those individual, within-movement adjustments disrupt long-term, pink-noise correlations associated with the time series of primary submovements—the initial open-loop component of the movement. In turn, such degradation of the movement-to-movement relations results in movement amplitude time-series whitening [6,7]. In addition to the ID-induced whitening of time-series structure, increases in mean movement time were induced [6, Fig 2; 7, Fig 4], which are thought to reflect the additional time needed for the operation of closed-loop visual feedback processes [e.g., 14].

Although it is reasonable to infer that pink-to-white-noise shifts in time-series structure reflect a transition from feedforward to feedback control, our aforementioned studies did not directly manipulate visual feedback availability. A manipulation of visual feedback would provide a more direct test of the hypothesis that shifts in time-series structure under visual feedback conditions reflect shifts in closed-loop motor control processes. For example, if increased closed-loop visual feedback processing is responsible for the ID-induced pink-to-white-noise shift in movement amplitude time series, then blocking visual feedback processing through the removal of visual feedback should result in increased dominance of feedforward control and a strengthened pink-noise time-series structure that remains constant across increases in the ID.

We are aware of one study that examined manual aiming movement amplitude time-series structure under both visual feedback and no-visual feedback conditions: In Miyazaki et al. [15, see Fig 1], long sequences of discrete elbow extensions were made over a distance of 45° to a point target. In that case, the task difficulty was very high [6, see Footnote 8]. According to the results of Miyazaki et al. [15], there was a pink-noise movement amplitude time-series structure when visual feedback was absent—when only vision of the target was available—and a white-noise time-series structure when the opportunity to engage visual feedback processing was available—when vision of both effector movement and the target was provided. However, we do not know how such a visual feedback manipulation would influence time-series structure under low-ID levels. Including both a low- and a high-ID level would provide a more complete assessment of how variations of visual feedback influence time-series structure. Relative to high-ID levels, we predict that varying the amount of visual feedback at low-ID levels should have little or no influence on time-series structure: At low-ID levels, performance success should largely depend on feedforward processing; closed-loop visual feedback processing should be minimally engaged, even when movement-related visual feedback is available [e.g., 14,16].

In the current experiment, participants performed in a cyclical aiming task under two ID levels—2 and 5 bits—and two visual feedback conditions—visual feedback and no-visual feedback, during which closed-loop visual feedback processing was enabled or disabled, respectively. Following our prior findings, under the visual feedback condition we predict a pink-to-white-noise shift in movement amplitude time-series structure with the increase in ID [6,7,17]. If the previously observed ID-induced whitening of time-series structure under visual feedback reflects engagement of closed-loop visual feedback processing, then preventing that processing in the no-visual feedback condition should result in strengthened pink noise that remains constant across the change in ID level. Alternatively, if the ID-induced pink-to-white-noise shift in time-series structure occurred in the no-visual feedback condition too, then a possible explanation of ID-induced whitening under visual feedback (and no-visual feedback) would be that it resulted from some change in open-loop, feedforward processing, or, possibly, an increase in closed-loop proprioceptive feedback processing [18–20]. An increase in the contribution of corrective sensory feedback processing from any modality (e.g., vision or proprioception) has the potential to increase time-series whitening. In sum, the current research will provide a more direct test of the influence of closed-loop visual feedback processing on cyclical aiming time-series structure under conditions where visual feedback processing should be less necessary (ID 2) and more necessary (ID 5).

## Materials and methods

### Participants

Thirty healthy individuals, 19 of whom were female, and whose mean age was 23.933 years (SD = 3.523), served as participants. All participants reported that they were right-hand dominant, had no prior history of neurological disease or damage, and had normal or corrected-to-normal vision. They responded to advertisements for healthy right-hand dominant volunteers between the ages of 18 and 30. The advertisements were flyers posted throughout the university. Upon completion of the experiment, 29 participants received a $10.00 USD payment and 1 participant received credit toward a course in which the student was enrolled. Each participant provided written informed consent that was approved by the Cleveland State University Institutional Review Board (29581-SLI-HS).

### Apparatus and procedure

The same apparatus and customized software reported in detail by Slifkin and Eder [7,21] was used in the current experiment and is briefly described here: Target displays were viewed on a vertically oriented flat-screen video monitor and movements were made on a horizontally oriented graphics tablet using its cordless mouse. Each target display consisted of two identical targets that were equidistant from the center of the monitor (e.g., see Fig 2). Using Fitts’ [9] equation for the ID—that is, ID = log_2_(2*A*/*W*)—we created two target displays, one that had an ID value of 2 bits (*A* = 15.88 mm, *W* = 7.94 mm) and one that had an ID value of 5 bits (*A* = 127 mm, *W* = 7.94 mm). With the increase in ID, there was an eight-fold increase in *A* while *W* was held constant. During the experiment itself, participants performed under both levels of ID twice, once under the visual feedback condition and once under the no-visual feedback condition. Thus, each participant performed in four unique experimental conditions. Each condition required the production of 500 consecutive cyclical aiming movements. (Additional information about our selection of ID levels and their component *A* and *W* values can be found in the “ID levels, scale levels, and future directions” section of the “Discussion.”)

#### Motor-output calibration period

As illustrated in the left panels of Fig 2, the visual information available on the video monitor was the same during the initial 100 movements of all four conditions: Participants were instructed that the position of a crosshairs *cursor* on the video monitor would correspond to the position of the mouse on the graphics tablet. They were told to move back and forth between the two targets, being as fast and accurate as possible—where the instruction to be fast was of equal importance to the instruction to be accurate—and that a target hit would register if the cursor “landed” anywhere within the target region at the time of a mouse-button press. For each movement, a crosshairs *marker* appeared at the center of the currently active target; when the mouse button was pressed to indicate the end of the action, the marker moved to the opposite target to mark it as the newly active target. Any button press occurring when the cursor was outside of the target was classified as a target miss and resulted in a “beep” sounded by the computer. The mouse cursor, targets, target marker, and error beep were active during the first 100 movements of all four experimental conditions (see Fig 2, left panels). That provided an initial period of calibration of motor output to the task requirements that was common to all four conditions.

#### Visual feedback and no-visual feedback conditions

The right panels of Fig 2 depict the available visual information over the remaining 400 movements: In the visual feedback conditions, the target marker and error “beep” were disabled, but both the mouse cursor and targets remained visible (Fig 2, top right panel). In the no-visual feedback conditions, the mouse cursor, target marker, and error “beep” were disabled, while the targets remained visible (Fig 2, bottom right panel). The critical difference between the visual feedback and no-visual feedback conditions was the presence and absence of the cursor, respectively. Under no-visual feedback, the removal of the cursor from the display disabled the operation of closed-loop visual feedback processing: It was no longer possible to use vision to monitor current cursor position relative to the target position—a capacity that remained intact in the visual feedback conditions. Participants were told that at some point in all conditions elements of the target display would change, but they should continue performing as they did before the change.

#### Practice conditions

Prior to the experiment itself and after an initial delivery of the task instructions and a demonstration of the task by the experimenter, participants practiced under each of the four experimental conditions. In all four practice conditions, a total of 100 movements was produced. The transition from the initial calibration period to the visual feedback or no-visual feedback conditions occurred after 50 movements. As was the case for the four experimental conditions, the order of each participant’s four practice conditions was randomized, with the exception that consecutive administration of the two ID-5 conditions was not permitted. That restriction was imposed to limit the buildup of fatigue that might accompany performance on consecutive ID-5 conditions. At the end of each experimental condition, participants were allowed to rest as long as they needed. Room lights were extinguished while the task was performed. In addition, a horizontally oriented black wooden board was fixed in position above the graphics tablet at, approximately, shoulder height. The board occluded participants’ view of their forearm and hand-mouse movements. Thus, the only task-related visual information available to participants was what was presented on the video screen. The total session duration was about 1 h.

#### Data acquisition

All data presented in this report came from the *x*-dimension of movement. The *x*-dimension control-to-display mapping was 1:1 such that a unit of mouse movement along the *x*-dimension of the graphics tablet translated to a unit of cursor movement along the *x*-dimension of the video display. Throughout each movement, data acquisition occurred every 15 or 16 ms (*M* ≈ 15.5 ms), which translates to instantaneous acquisition rates of either 66.67 or 62.50 Hz (*M* ≈ 64.52 Hz), respectively. The spatial resolution of each sample was 0.1 mm. In addition, during data acquisition we recorded the *x*-dimension movement-trajectory position at the moment of each mouse-button press.

### Experimental design and data analyses

#### General data processing

The main analysis involved spectral analysis of time series of consecutive movement amplitude measurements, where each movement amplitude measurement was the *x*-axis distance between consecutive mouse-button press positions. We did not analyze the initial 200 movements of the time series in any experimental condition. That excluded the initial 100-movement calibration period (movements 1–100) and the first 100 movements following the transition (movements 101–200). The latter was meant to exclude any transient adjustments that may have been present following the transition. The remaining 300 movements in each condition (movements 201–500) for each participant were submitted to data analyses. (For one participant in the ID-5-visual-feedback condition, movements 200–499 were submitted to data analyses; movement 500 was removed because its movement time value was an extreme outlier.)

#### Mean movement time and mean movement amplitude

While analysis of movement amplitude time-series structure via spectral analysis was the primary focus of the current study, we also present an analysis of mean movement amplitude and mean movement time; the latter was the average time from one mouse-button press to the next. Analysis of those variables allowed an assessment of whether the ID manipulation was effective, which should be evident in between-ID differences for movement amplitude and movement time. For example, the analysis of mean movement amplitude allowed a determination of the scaling of movement amplitude to the different amplitude requirements imposed under ID 2 and ID 5. Following Fitts’ law, its numerous replications, and our prior research, it was expected, at least under visual feedback conditions, that movement time would increase with the increase in ID [e.g., 6,7,9,13,14,21]. In addition, the analysis of movement time can provide information about the extent to which closed-loop visual feedback processes were engaged in the control of movement amplitude. That is, under conditions of visual feedback and instructions to maximize movement speed and accuracy, increases in movement time beyond a minimum value [e.g., ≈ 200 ms: 10,22] correlate with increased engagement of closed-loop visual feedback processes [e.g., 4,23,24]. Previously, under visual feedback conditions we found that increases in ID induced parallel increases in movement time and time-series whitening [6,7]. Those findings suggested that closed-loop visual feedback processing related to movement time lengthening might also be related to time-series whitening [7].

The average movement amplitude and movement time were calculated for each participant’s time series under each of the four unique conditions. Separate two-way ID (2) by visual feedback (2) ANOVAs, with repeated measures on both factors, were used to test the reliability of the change in mean movement amplitude and mean movement time with the change in ID and visual feedback conditions.

#### Spectral analysis of movement amplitude time series

Assessments of movement amplitude time-series structure were made through spectral analysis. Prior to spectral analysis, each 300-point movement amplitude time series was linearly detrended [e.g., 8]. The detrending involved calculating the best-fitting linear regression for each time series and obtaining the time-series residuals. That process removed the mean and any overall linear trend in the time series, but otherwise preserved the original time-series structure. Next, the power spectral density for each residual time series was calculated in Matlab v.7.1 using Welch’s averaged-modified-periodogram method, that is, Matlab’s *pwelch* function. We chose *pwelch* parameter values that allowed each series to be analyzed as a single segment (window) of 300 data points. (In particular, the *pwelch* parameter values were [a] *window* = 300, [b] *noverlap* = 0, [c] *nfft* = 300, and [d] *fs* = 1.) Then, the 300-point segment was multiplied by a Hamming window followed by the calculation of the power spectral density. The power spectrum from each time series was divided into 150 bins of equal width where the frequencies associated with the upper limits of the lowest and highest frequency bins were 0.0033 and 0.5000 cycles per movement (Hz), which translates to 300 and 2 movements per cycle, respectively. Each frequency bin spanned a 0.0033 Hz range. The amount of power in each frequency bin was related to the magnitude of the movement amplitude oscillations within that bin’s frequency range.

**Fig 3:**
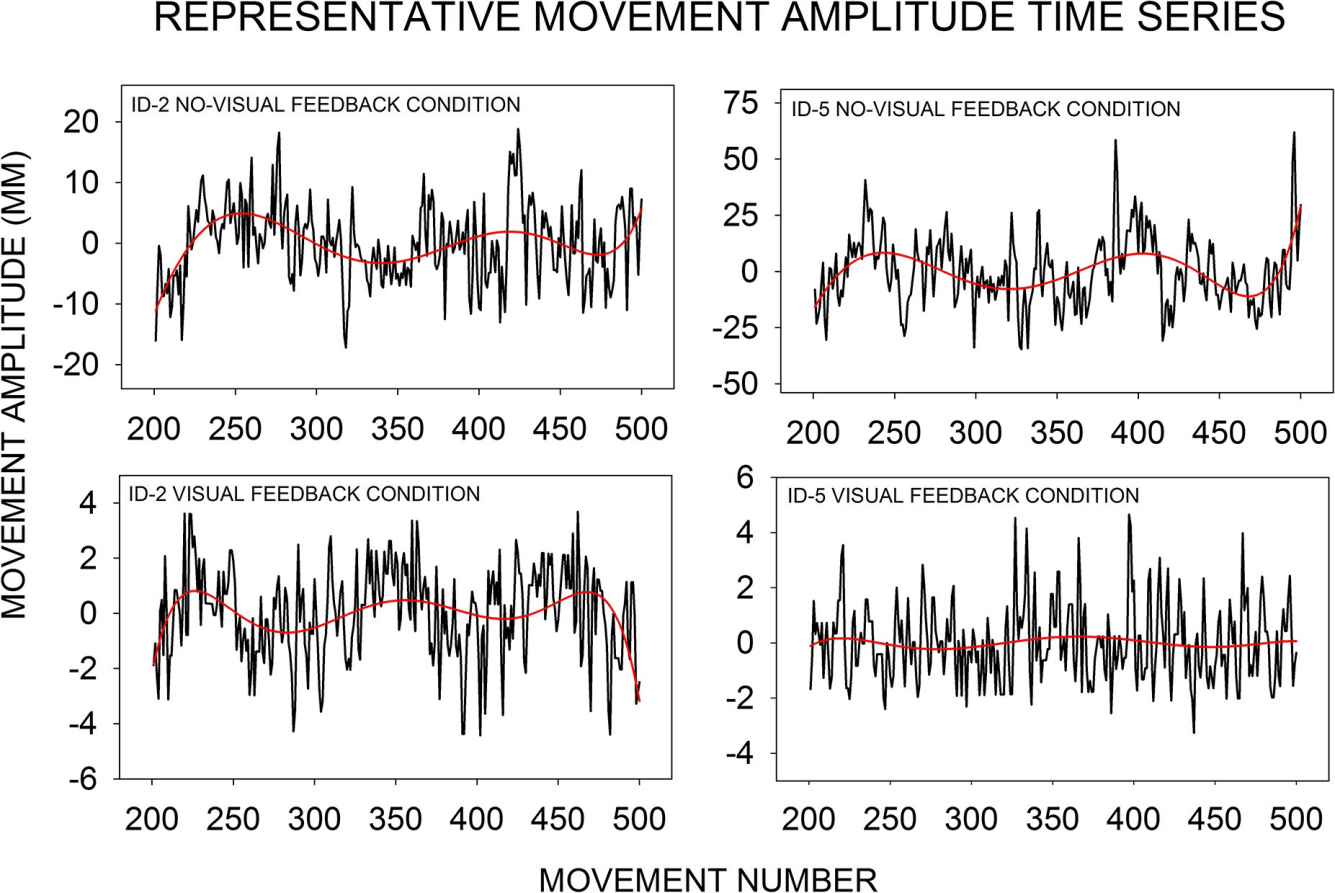
Representative movement amplitude time series from the four experimental conditions. The time series for ID-2 no-visual feedback (top left panel), ID-5 no-visual feedback (top right panel), ID-2 visual feedback (bottom left panel), and ID-5 visual feedback (bottom right panel) were taken from Participant 29, 17, 30, and 5, respectively. As with all data submitted to spectral analysis in the study, each panel in the figure contains a linearly detrended (and demeaned) movement amplitude time series from the last 300 consecutive movements in each condition (see ‘‘Spectral analysis of movement amplitude time series”). As one means of highlighting across-condition changes in movement amplitude time-series structure—namely, the contribution of low-frequency wavelike oscillations—sixth-order polynomial regression equations were fit to each time series.

Our main measure of movement amplitude time-series structure was an estimate of *β* from the power spectrum. That involved a log_10_ transformation of both the spectral power and frequency of each participant’s power spectrum in each condition. Then, linear regression—*y* = *bx* + *a*—was used to describe changes in log_10_ power as a function of log_10_ frequency (see Fig 1, right panels). Prior to fitting the linear regression, following Van Orden et al. [25], the lowest frequency bin (with an upper limit of 0.0033 Hz or −2.4771 Hz after the log_10_ transformation) was removed from the analysis. That step was taken because the aforementioned linear time-series detrending procedure has been shown to attenuate power in the lowest frequency bin, which may bias estimates of *β* [see 25, pp. 117–120]. Thus, the linear regression equation spanned the lowest remaining frequency bin, which had an upper limit of 0.0067 Hz—or −2.1761 Hz after the log_10_ transformation—to the frequency bin with an upper limit of 0.1000 Hz—or −1.0000 Hz after the log_10_ transformation. The reason for imposing a −1.0000 Hz upper limit is that a common feature of human performance time-series data exhibiting long-range correlations (including the current data) is a linear reduction in power across the lower frequencies until about −1.0000 Hz, after which there is a flattening, a sharp downturn, or a sharp upturn in power across the higher frequencies [e.g., see 6, Footnote 6; 8, Fig 13: interval timing, & Fig 14: force production, angular rotation, manual aiming; 26, Fig 3: manual aiming; 27, Fig 2: interval timing]. Thus, assessments of the lower frequency region of the power spectrum allow for clearer distinctions between different forms of colored noise, for example, pink vs. white noise.

The negative of the linear regression slope *b* describing the relation between log_10_ frequency and log_10_ power is equivalent to the exponent *β* in the equation for the power function S(*f*) = 1/*f*^*β*^. In that equation, *S*(*f*) is the spectral power at each frequency, 1/*f* is the inverse of frequency, and *β* specifies the degree of proportionality between *S*(*f*) and 1/*f* [e.g., see 28, p. 455]. In other words, if the linear regression *b* has a value of −1, then the power function *β* has a value of 1. If *β* = 1, then the fall-off of power is inversely proportional to the increase in frequency, which is the signature of a pink-noise time-series structure (e.g., see Fig 1, bottom right panel). If *β* = 0, then there is no change in power as a function of frequency, that is, the spectrum is flat, which is the signature of a white-noise time-series structure (e.g., see Fig 1, top right panel).

A two-way ID (2) by visual feedback (2) ANOVA, with repeated measures on both factors, was used to examine changes in the group-mean *β* with the change in ID and visual feedback conditions. (When separate two-way ANOVAs included estimates of *β* that either retained or excluded the lowest spectral frequency bin, the between-ANOVA statistical-outcomes [statistically significant or not] were the same for each main effect and the interaction.) For each dependent variable—mean movement amplitude, mean movement time, and *β*—the Tukey HSD post-hoc test was used to compare pairs of group means, which allowed us to investigate the locus of any significant main effects or interactions. All results reported as “significant” had *p* values less than .05. Exact *p* values were reported, unless the *p* value was less than .0001; in that case, *p* < .0001 was reported. All statistical analyses were performed using Statistica v.13.3.

## Results

For three participants, data from 1 of the 4 experimental conditions was incomplete or missing. In two cases, the data files were corrupted. In the third case, one of the four conditions was run twice, which resulted in the absence of data for one condition. Two solutions were considered: The first solution entailed removing all data of the three participants from all analyses; the second solution entailed replacing the missing mean movement amplitude, mean movement time, and *β* values with an average across the remaining participants within the relevant condition. When separate two-way ANOVAs based on the two solutions were compared, the between-ANOVA statistical outcomes (statistically significant or not) were the same for each dependent variable’s main effects and interaction. Furthermore, the two analyses applied to each dependent variable yielded essentially identical patterns of group means. Here, we report the results based on the missing data replacement procedure. Before reporting the statistical analyses of our results, we present representative time series from each condition.

### Representative movement amplitude time series

As with all data submitted to spectral analysis in the study, each panel in Fig 3 contains a linearly detrended (and demeaned) movement amplitude time series, where each time series contains the last 300 consecutive movements in each condition (see ‘‘Spectral analysis of movement amplitude time series”). Each panel in Fig 3 plots a time series from a representative participant in each of the four experimental conditions: ID-2 no-visual feedback (top left panel, Participant 29), ID-5 no-visual feedback (top right panel, Participant 17), ID-2 visual feedback (bottom left panel, Participant 30), and ID-5 visual feedback (bottom right panel, Participant 5). In addition, as one way of highlighting the presence of low-frequency wavelike oscillations, sixth-order polynomial regression functions were fit to each time series. A reduction in the amplitude of low-frequency time-series oscillations may be correlated with a flattening of the power spectrum and therefore time-series whitening (see Fig 1).

As shown in the time series and as highlighted by the polynomial regression functions, the contribution of low-frequency oscillations appears to be similar across the ID-2 no-visual feedback (top left panel), ID-5 no-visual feedback (top right panel), and ID-2 visual feedback (bottom left panel) conditions, but at the ID-5 visual feedback condition (bottom right panel) the polynomial regression function has flattened. In particular, at ID 2 a similar low-frequency-dominant time-series structure can be seen either when visual feedback was absent or present (cf. top left panel and bottom left panel). However, at ID 5 the low-frequency-dominant structure was attenuated when visual feedback was added (cf. top right panel and bottom right panel). It is at ID 5 where the control of manual aiming should require greater reliance on visual feedback processing and there should be increased time-series whitening. While the main results of this study will focus on further analyses of movement amplitude time-series structure (via spectral analysis), prior to that presentation we provide an analysis of the group-mean movement amplitude and the group-mean movement time.

### Movement amplitude

**Fig 4:**
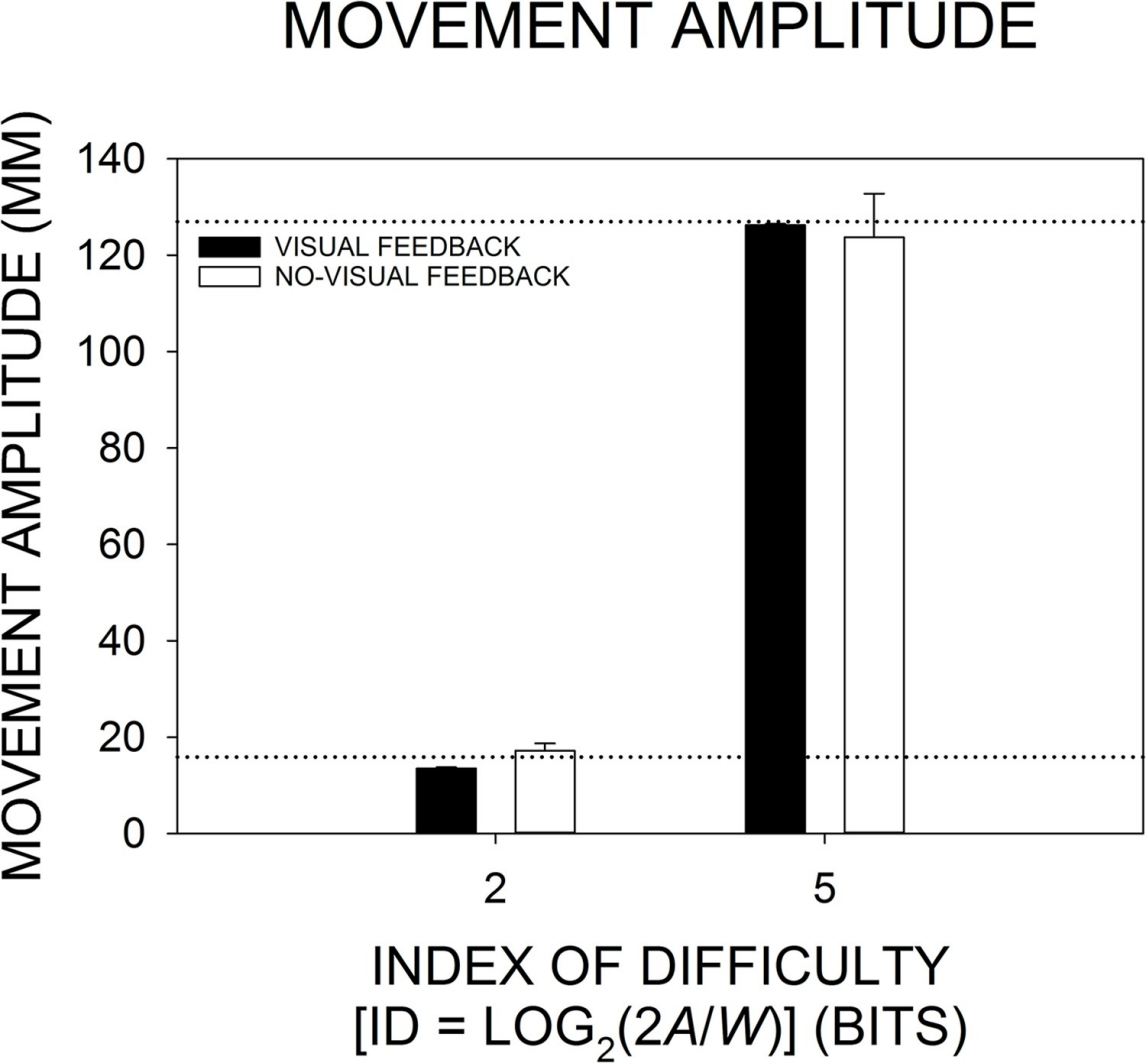
Changes in the group-mean movement amplitude as a function of the index of difficulty (ID) and visual feedback condition. The height of each bar represents the group-mean movement amplitude based on an across-participant average at each ID and visual feedback condition. The *error bar* extending above each group-mean bar represents 1 SEM. The upper and lower horizontal-dotted lines mark the movement amplitude requirements under ID 2 (15.88 mm) and ID 5 (127 mm), respectively.

As shown in Fig 4, as the ID and, therefore, the movement amplitude requirement increased, the group-mean movement amplitude increased. When averaged across visual feedback conditions within each ID level, the group-mean movement amplitude at ID 2 and ID 5 was 15.355 mm and 125.004 mm, respectively. Each group mean closely matched its corresponding movement amplitude requirement. The increase in movement amplitude with the increase in ID resulted in a significant main effect of ID, *F*(1, 29) = 634.297, *p* < .0001, *η*_*p*_^2^ = .956. When averaged across ID levels within each visual feedback condition, the group-mean movement amplitude was 69.892 mm and 70.466 mm in the visual feedback and no-visual feedback conditions, respectively. Those values were nearly identical, resulting in the absence of a significant main effect of visual feedback, *F*(1, 29) = 0.014, *p* = .907, *η*_*p*_^2^ = .0005. As seen in Fig 4, the parallel ID-induced increases in the group-mean movement amplitude under visual feedback and no-visual feedback led to the absence of a significant ID by visual feedback interaction, *F*(1, 29) = 0.552, *p* = .464, *η*_*p*_^2^ = .019.

According to the Tukey HSD post-hoc test, there was a significant increase in the group-mean movement amplitude both from ID-2 visual feedback to ID-5 visual feedback (*p* = .0002) and from ID-2 no-visual feedback to ID-5 no-visual feedback (*p* = .0002). Differences between group means were not significant for either the ID-2-visual-feedback vs. ID-2-no-visual-feedback comparison (*p* = .924) or the ID-5-visual-feedback vs. ID-5-no-visual-feedback comparison (*p* = .973). (There were reliable increases in the group-mean movement amplitude for both the change from ID-2 no-visual feedback to ID-5 visual feedback [*p* = .0002] and from ID-2 visual feedback to ID-5 no-visual feedback [*p* = .0002].) The pattern of Tukey HSD results reflects both the ANOVA results and the pattern of group means depicted in Fig 4.

### Movement time

**Fig 5:**
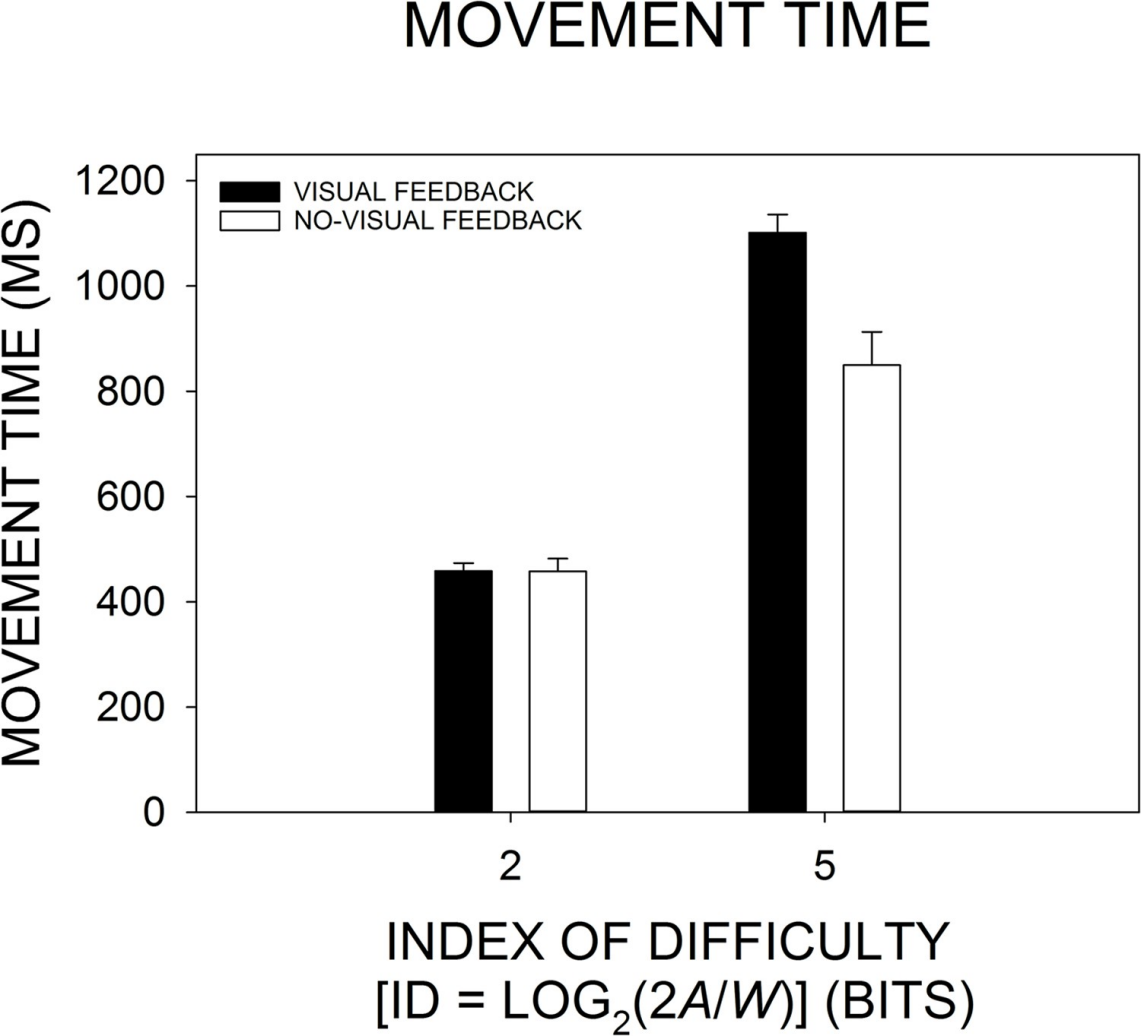
Changes in the group-mean movement time as a function of the index of difficulty (ID) and visual feedback condition. The height of each bar represents the group-mean movement time based on an across-participant average at each ID and visual feedback condition. The *error bar* extending above each group-mean bar represents 1 SEM.

As shown in Fig 5, as might be anticipated from Fitt’s law [e.g., 9], movement time increased with the increase in ID: When averaged across the two visual feedback conditions within each ID level, the group-mean movement time at ID 2 and ID 5 was 457.999 and 975.797 ms, respectively. That increase was reflected by a significant main effect of ID, *F*(1, 29) = 273.108, *p* < .0001, *η*_*p*_^2^ = .904. When averaged across ID levels within each visual feedback condition, movement time under visual feedback and no-visual feedback was 780.100 ms and 653.696 ms, respectively. The reduction in movement time with the removal of visual feedback resulted in a significant main effect of visual feedback, *F*(1, 29) = 11.326, *p* = .002, *η*_*p*_^2^ = .281. However, the magnitude of that effect differed at the different ID levels (see Fig 5): At ID 2, movement time was essentially the same under the two visual feedback conditions (ID-2 visual feedback = 458.416 ms, ID-2 no-visual feedback = 457.582 ms), but the ID-2-to-ID-5 increase in movement time was greater under visual feedback (ID 5 = 1101.783 ms) than no-visual feedback (ID 5 = 849.810 ms). That gave rise to an ID-5 no-visual-feedback-to-visual-feedback increase of 251.973 ms. The increased influence of the visual feedback manipulation as ID increased yielded a significant ID by visual feedback interaction, *F*(1, 29) = 17.163, *p* = .0003, *η*_*p*_^2^ = .372.

According to the Tukey HSD post-hoc test, there was a significant increase in the group-mean movement time both from ID-2 visual feedback to ID-5 visual feedback (*p* = .0002) and from ID-2 no-visual feedback to ID-5 no-visual feedback (*p* = .0002). While a statistically significant difference between ID-2 visual feedback and ID-2 no-visual feedback was absent (*p* = 0.999), there was a significant increase of the ID-5-visual-feedback movement time over the ID-5-no-visual-feedback movement time (*p* = .0002). That provides further confirmation of the larger ID-induced increase in movement time under visual feedback as compared with no-visual feedback. (There were reliable increases in movement time for both the change from ID-2 no-visual feedback to ID-5 visual feedback [*p* = .0002] and from ID-2 visual feedback to ID-5 no-visual feedback [*p* = .0002].) The pattern of Tukey HSD results reflects both the ANOVA results and the pattern of group means depicted in Fig 5.

**Fig 6:**
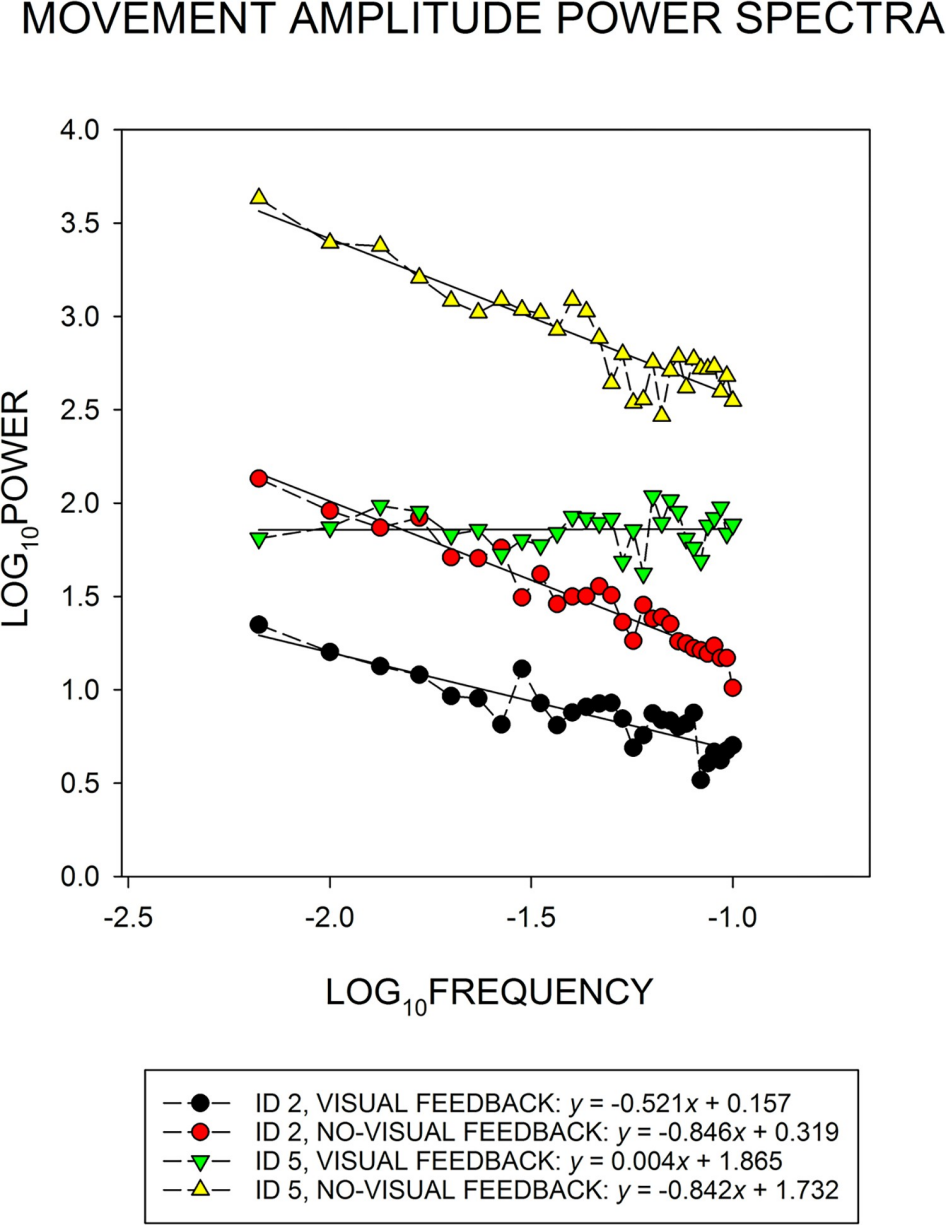
Changes in the group-mean power spectrum across index of difficulty (ID) and visual feedback conditions. Each data point within each spectrum represents the across-participant average of log_10_ power within a given log_10_ frequency bin. The negative of the slope (−*b*) of the linear regression equation—*y* = *bx* + *a*—fit to each group-mean power spectrum was equivalent to the exponent (*β*) of a power function—*y* = 1/*x*^*β*^—that is, if *b* = −1, then *β* = 1. *β* was the primary index of movement amplitude time-series structure. The amount of spectral power within each frequency bin represents the magnitude (size) of the time-series oscillations or variability within that frequency bin. In turn, the elevation of each spectrum represents the amount of overall movement amplitude variability across the range of frequencies.

**Fig 7:**
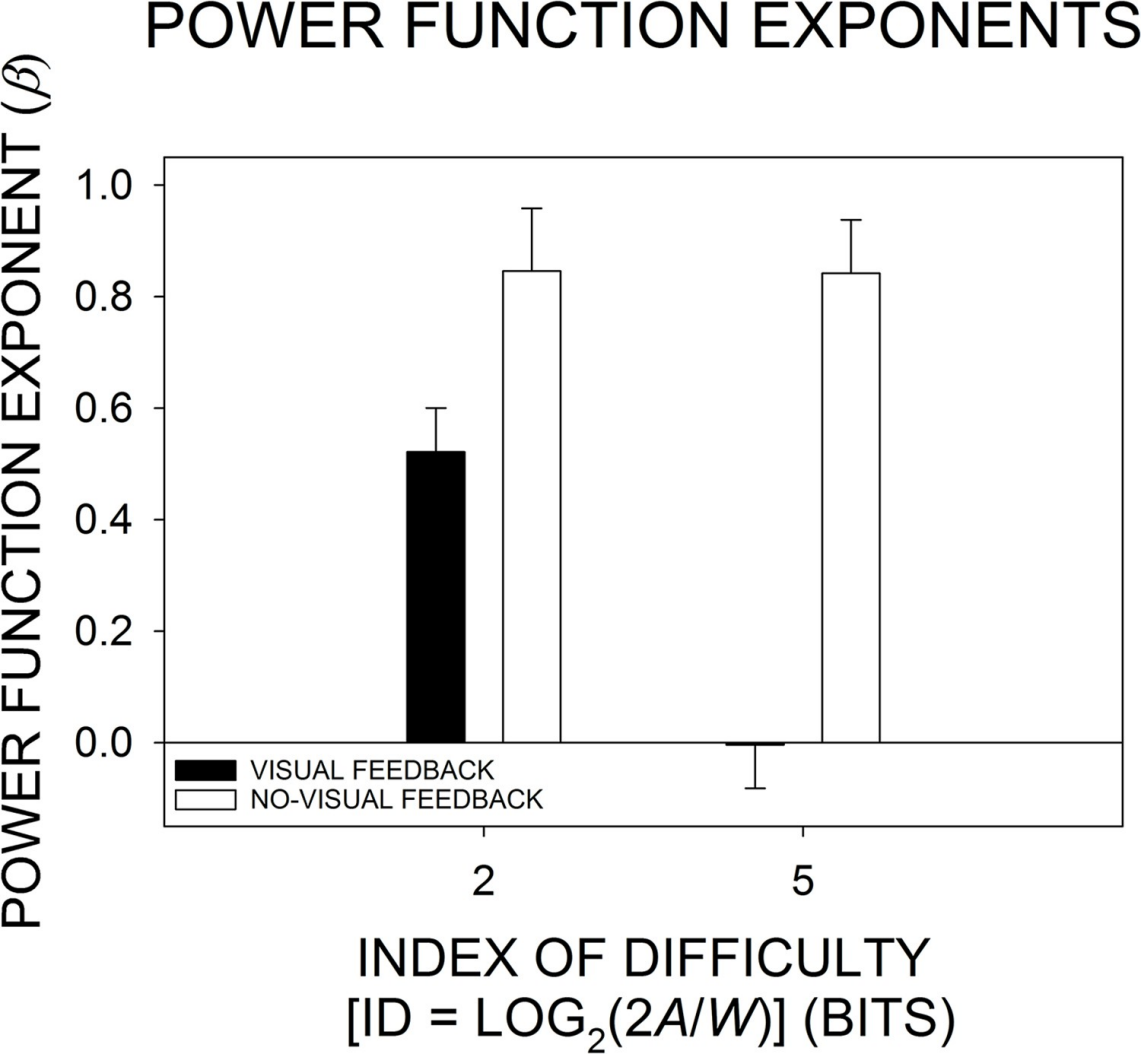
Changes in the group-mean *β* as a function of the index of difficulty (ID) and visual feedback condition. *β* is the exponent of a power function—*y* = 1/*x*^*β*^. A *β* value was calculated for each participant’s power spectrum at each ID level and visual feedback condition. Then, each group-mean *β* value was calculated by averaging across the individual-participant *β*s at a given ID and visual feedback condition. The *error bar* extending above each group-mean bar represents 1 SEM.

Fig 6 shows the group-mean movement amplitude power spectrum at each ID and each visual feedback level. For each spectrum, linear regression equations provided a nice description of changes in log_10_ power as a function of log_10_ frequency. Under visual feedback, the slope (*b*) of the regression line is negative at ID 2 and clearly flattens to a near-zero value at ID 5. In contrast, under no-visual feedback, the regression *b*s are negative and parallel for the ID-2 and ID-5 power spectra. That pattern of regression *b* results based on the group-mean power spectra matched the pattern of group-mean *β* coefficients plotted in Fig 7: When visual feedback was available, *β* was reduced from 0.521 to −0.004 with the change from ID 2 to ID 5; thus, the distribution of power was in the pink-noise range at ID 2 and clearly whitened at ID 5. However, under no-visual feedback, *β* was essentially unchanged with the increase in ID, that is, *β* at ID 2 and ID 5 was 0.846 and 0.842, respectively; thus, in the absence of visual feedback, the distribution of power exhibited a strengthened pink-noise structure that persisted with the change in ID level.

When averaged across visual feedback conditions within each ID level, *β* declined from 0.684 at ID 2 to 0.419 at ID 5. The overall ID-induced decline in *β* was reflected by a significant main effect of ID, *F*(1, 29) = 9.807, *p* = .004, *η*_*p*_^2^ = .253. When averaged across ID levels within each visual feedback condition, *β* increased from 0.259 under visual feedback to 0.844 under no-visual feedback, which yielded a significant main effect of visual feedback, *F*(1, 29) = 38.164, *p* < .0001, *η*_*p*_^2^ = .568. The differential influence of the visual feedback manipulation on the ID-induced change in *β*—that is, the ID-2-to-ID-5 reduction in *β* under visual feedback and the absence of an ID-induced change in *β* under no-visual feedback—resulted in a significant ID by visual feedback interaction, *F*(1, 29) = 6.323, *p* = .018, *η*_*p*_^2^ = .179. Under visual feedback, the ID-induced change from pink to white noise closely replicates our prior findings: In the current study (Fig 7), Slifkin and Eder [6, Fig 3], and Slifkin and Eder [7, Fig 6], at ID 2 the group-mean *β* was 0.521, 0.50, and 0.41, and at ID 5 the group-mean *β* was −0.004, 0.09, and 0.03, respectively. In contrast, as predicted, under no-visual feedback there was strengthened pink noise that remained constant over the increase in ID.

The Tukey HSD post-hoc test confirmed, namely, that the reduction in the group-mean *β* from ID-2 visual feedback to ID-5 visual feedback was significant (*p* = .006), but the very small difference between ID-2 no-visual feedback and ID-5 no-visual feedback was not significant (*p* = 0.999). In addition, the reduction in the group-mean *β* from ID-2 no-visual feedback to ID-2 visual feedback was not significant (*p* = .143), whereas the reduction from ID-5 no-visual feedback to ID-5 visual feedback was significant (*p* = .0002). (The reduction from ID-2 no-visual feedback to ID-5 visual feedback was reliable [*p* = .0002], but the increase from ID-2 visual feedback to ID-5 no-visual feedback was not reliable [*p* = .151].) The pattern of Tukey HSD results reflects both the ANOVA results and the pattern of group means depicted in Fig 7.

## Discussion

Our earlier research showed that movement amplitude time-series structure in a cyclical aiming task shifted from pink to white noise with increases in the ID [6,7]. In that research, we suggested that the increase in ID induced engagement of closed-loop visual feedback processing. Further support for that hypothesis was provided by a computer simulation model fit to the data of Slifkin and Eder [6]. The model included 1) a primary submovement—responsible for the initial distance covering portion of the trajectory—with a time series that oscillated with a pink-noise structure, and 2) a secondary submovement that would only be engaged if the primary submovement endpoint was destined to terminate outside the target boundaries; the secondary submovement time series oscillated with a white-noise structure. According to the model, when the ID was low, engagement of the feedforward, primary submovement was typically sufficient for achieving high levels of aiming accuracy. That gave rise to a pink-noise time-series structure. However, as difficulty increased it was more likely that movement accuracy would require the addition of a corrective secondary submovement. In the model, those adjustments to individual primary submovements degraded movement-to-movement relations underlying the primary submovement’s pink-noise time-series structure. Like the empirical data, the simulated time series whitened with increases in the ID [6, see Fig 6].

The current study provided a more direct test of the hypothesis that increased whitening of movement amplitude time series reflects increased engagement of closed-loop visual feedback processes [6,7,17]. If that hypothesis is correct, then it follows that disabling closed-loop visual feedback processes should result in strengthened pink-noise time series that should not change with the increase in ID. Indeed, under visual feedback we replicated our prior findings that increases in ID induce pink-to-white-noise shifts in movement amplitude time-series structure [6,7], and in the current no-visual feedback condition we found strengthened pink noise that was unchanged with the increase in ID (see Fig 7). Thus, there is support for the claim that whitening of time-series structure under visual feedback conditions reflects ID-induced engagement of closed-loop visual feedback processing. Had there been ID-induced whitening of time-series structure under both visual feedback and no-visual feedback, then a possible explanation of the ID-induced whitening would be that it resulted from some change in open-loop processing, or, possibly, an increase in closed-loop proprioceptive processing [18–20]. Although an increase in the contribution of corrective sensory feedback processing from any modality (e.g., vision or proprioception) has the potential to increase time-series whitening, the strengthened pink noise observed under the ID-5 no-visual feedback condition (vs. the white noise observed under the ID-5 visual feedback condition) suggests that any existing proprioceptive feedback processing did not have enough influence on movement amplitude control to result in increased time-series whitening.

When viewing the results as changes across visual feedback conditions within each ID level, we also found confirmation of our predictions: Within ID 2, the group-mean *β* values for the no-visual feedback and visual feedback conditions were in the pink-noise range and did not differ statistically. That finding is consistent with the idea that at low-ID levels there is little if any need to engage closed-loop visual feedback processing, even when visual feedback is available [e.g., 14]. In contrast, within ID 5 there was a clear and reliable pink-to-white-noise shift with the change from no-visual feedback to visual feedback (a result matching the discrete aiming findings of Miyazaki et al. [15]). That shift could be attributed to the need to engage visual feedback processing when visual feedback was available [e.g., 14].

### Movement time and movement amplitude

As might be predicted by Fitts’ law and its numerous replications [e.g., 9,13], we found that movement time increased with the increase in ID not only in the visual feedback condition but in the no-visual feedback condition too. However, as seen in Fig 5, those ID-induced increases were larger under visual feedback than no-visual feedback: At ID 2, movement time was essentially the same under both visual feedback conditions, but at ID 5 movement time in the visual feedback condition was 251.973 ms greater than in the no-visual feedback condition. If a source of movement time lengthening is the engagement of visual feedback [e.g., 4,10,14,22–24], then it follows that movement time should be longer under the ID-5-visual-feedback condition—where it was possible to engage time-consuming visual feedback processes—and shorter under the ID-5-no-visual-feedback condition—where engagement of visual feedback processes was blocked.

Furthermore, the current pattern of movement time results is consonant with the findings of Wu et al. [29]. They examined performance in a discrete manual aiming task under ID 1, 2, 3, 4, 5, and 6 bits and under different conditions of visual information availability: “full vision,” “no target,” “no movement,” and “no vision.” Their full vision and no movement conditions were equivalent to the current visual feedback and no-visual feedback conditions, respectively. Wu et al. [29] found no differences in the group-mean movement time across all visual information conditions within each of the first four ID levels. However, within both ID 5 and 6, movement time under full vision was significantly longer than that recorded in the other visual information conditions, including their no movement condition, which, again, is equivalent to the current no-visual feedback condition. In addition, Meyer et al. [4] examined performance in a discrete wrist rotation task when the target was always visible but a cursor representing wrist position was either visible or invisible, which were conditions analogous to the current visual feedback and no-visual feedback manipulations, respectively. When we plotted the movement time data from the visible and invisible cursor conditions of Meyer et al. [4, see Tables 4 & 5, pp. 355 & 359], a pattern of results matching the Wu et al. [29] results could be seen [see also 18, Fig 1]. Thus, the pattern of previously reported movement time results matches the current results: Here, at a low-ID level (2 bits) there was no movement time difference between the visual feedback and no-visual feedback conditions, but at a high-ID level (5 bits) movement time for the visual feedback condition exceeded that of the no-visual feedback condition (Fig 5).

As shown in Fig 4, the group-mean movement amplitude was precisely scaled to the ID-2 and ID-5 amplitude requirements under both visual feedback and no-visual feedback (a finding in accord with Berkinblit et al. [30, see Fig 1] who included feedback conditions like those of the current study). However, it may be of interest to note that the between-participant variability increased with the change from visual feedback to no-visual feedback. As reflected by the size of the SEM error bars in Fig 4, the individual-participant mean movement amplitudes were tightly clustered and close to the movement amplitude requirement at both ID levels under visual feedback, but the width of the distribution grew when visual feedback was removed, particularly at ID 5: When visual feedback was removed, there was a 6.461-fold increase in the SEM at ID 2, and a 33.954-fold increase in the SEM at ID 5. As compared with visual feedback, under no-visual feedback some participants maintained similar mean movement amplitudes while others produced larger or smaller mean movement amplitudes. The growth in between-participant variability may reflect participants’ inability to engage corrective, closed-loop visual feedback processing when visual feedback was absent. In the absence of such information processing, the source of the increased variability could be individual differences in the amplitude setting of the uncorrected feedforward, open-loop component of movement, that is, the primary submovement.

An initial, main purpose of including analyses of the mean movement time and movement amplitude was to check the effectiveness of the ID manipulation, to determine if the increase in ID induced the expected increase in both movement time and movement amplitude. Indeed, there were reliable ID-induced increases in both dependent variables under both visual feedback and no-visual feedback. That is, participants always treated the two ID levels as being distinctly different. Because of our own research and the extensive research on manual aiming under visual feedback conditions [e.g., 6,7,9,13,14,21], we were confident that participants would discriminate between ID 2 and ID 5 under the current visual feedback condition. However, we were less confident that would be the case under no-visual feedback. Had participants adopted common movement time and common movement amplitude values under both ID levels in the no-visual feedback condition, then it would have been difficult to attribute the nearly identical ID 2 and ID 5 *β* values to the absence of visual feedback (Fig 7). Rather, the similarities in *β* could have resulted from participants operating under ID levels that were effectively the same. However, again, that was not an issue as our analyses of movement time and movement amplitude showed that participants always made a clear distinction between the two ID levels (see Figs 4 & 5).

### Sensitivity to closed-loop visual feedback processing: Movement time vs. *β*

As previously reviewed, movement time lengthening is often thought to reflect engagement of visual feedback processes. However, the primary submovement—primarily reflecting the operation of feedforward control—has been shown to increase in duration with increases in the ID, both when visual feedback was present and when it was absent [11,12: feedback present; 4: feedback present or absent]. In the no-visual feedback condition of the current study, we assume the movement time values at ID 2 and ID 5 reflect operation of the primary submovement alone. We also assume that under visual feedback the primary submovement contributes the same amount of time to the total movement time as in the no-visual feedback condition. In that case, as compared with the ID-2-to-ID-5 increase in movement time in the no-visual feedback condition (457.582 to 849.810 ms, a 392.228-ms increase), the additional ID-2-to-ID-5 increase in movement time in the visual feedback condition (458.416 to 1101.783 ms, a 643.367-ms increase) should reflect the contribution of secondary submovements (643.367 ms − 392.228 ms = 251.139 ms) (see Fig 5). Thus, the increase in total movement time was sensitive to the increase in ID, but, even in the visual feedback condition, it appears that primary submovement duration was more sensitive to the increase in ID than was secondary submovement duration.

In contrast, there was no ID-induced change in *β* under no-visual feedback, but a strong ID-induced reduction in *β* (i.e., whitening) under visual feedback. Thus, while movement time was always sensitive to variations in ID, *β* was only sensitive to variations in ID when visual feedback was available and there was an ID-induced need to engage closed-loop visual feedback processing. How can those findings be explained in terms of our model of movement amplitude time-series structure [6,7]? According to that model, when task constraints are constant within conditions, the time series of primary submovement amplitudes will always have a pink-noise structure that does not change in response to between-condition variations in task constraints—for example, movement amplitude requirement, target width, ID, or even the presence or absence of visual feedback—or any resultant variations in primary submovement kinematics—for example, duration, amplitude, or velocity. Therefore, it is only through the addition of secondary submovements—a product of closed-loop visual feedback processing—that the structure of movement amplitude time series will change, that is, whiten. The exclusive relation between variations of movement amplitude time-series structure and closed-loop visual feedback processing explains both 1) *β*’s insensitivity to the increase in ID in the absence of visual feedback—when visual feedback processing was disabled—and 2) *β*’s sensitivity to the increase in ID in the presence of visual feedback—when visual feedback processing was enabled.

Slifkin and Eder [7, see Figs 4 & 7] found that under visual feedback conditions the ID was a strong predictor of both the group-mean movement time and the group-mean *β*. In turn, there was a strong relation between variations of *β* and variations of movement time, *r*^2^ = .981 [7, p. 1660]. Because variations in movement time are thought to reflect variations in the amount of closed-loop visual feedback processing [e.g., 4,10,14,22–24], we concluded that movement time’s high shared variance with *β* must reflect *β*’s dependence on closed-loop visual feedback processing too [7]. A refinement in our current thinking is that under visual feedback conditions it is only the portion of the ID-induced increase in total movement time resulting from closed-loop visual feedback processing that gives rise to movement amplitude time-series whitening. A larger portion of the ID-induced increase in movement time, associated with primary submovement lengthening, does not appear to contribute to time-series whitening. When the relation between the group-mean movement time and *β* from the four conditions of the current experiment was examined, the *r*^2^ value was more than halved, *r*^2^ = .413, compared to the same relation examined in Slifkin and Eder [7] when visual feedback was always available, *r*^2^ = .981. The reduction in the current *r*^2^ value occurred because, although under visual feedback the increase in movement time was related to a decrease in *β*, under no-visual feedback the increase in movement time was related to no change in *β* (cf. Figs 5 & 7). We believe that the absence of the relation between movement time and *β* under no-visual feedback resulted from the disengagement of corrective secondary submovements. Perhaps counterintuitively, the reduced *r*^2^ value in the current study provides strengthened support for the notion that ID-induced whitening under visual feedback results from engagement of closed-loop visual feedback processes.

### ID levels, scale levels, and future directions

Would the current results have differed if the 2-to-5-bit increase in the ID was achieved through a reduction in target width while the movement amplitude requirement was held constant? In our prior research, under conditions of visual feedback we examined changes in movement time and *β* as ID increased from 2-to-5 bits both under a set of *small-scale* (2 bits: *A* = 63.5 mm, *W* = 31.75 mm; 5 bits: *A* = 63.5 mm, *W* = 3.97 mm) and under a set of *large-scale* (2 bits: *A* = 127 mm, *W* = 63.5 mm; 5 bits: *A* = 127 mm, *W* = 7.94 mm) target displays [7, see Fig 1]. Within each scale level, the 2-to-5-bit increase in ID was achieved through an eight-fold decrease in target width while the movement amplitude requirement remained constant; within each ID level, from the small- to large-scale level, there was a doubling of both the movement amplitude requirement and target width values, that is, target display *scale* doubled. We found that with the increase in the ID, movement time increased and *β* changed from pink-to-white-noise values (those outcomes were observed in the visual feedback condition in the current study too [see Figs 5 & 7]). Furthermore, for both movement time and for *β* there were no statistically significant differences between scale levels within either the 2-bit or 5-bit ID level [7, see Figs 4 & 6]. Thus, in the current study, had we included a 2-to-5 bit increase in ID achieved through a reduction in target width with the movement amplitude requirement held constant, we believe the same basic pattern of results would have been found as reported here, where the increase in ID was achieved through an eight-fold increase in the movement amplitude requirement with target width held constant. As opposed to the absolute value of either the movement amplitude requirement or target width alone, the ID is the more parsimonious predictor of movement time and *β* [7].

One might also wonder whether including additional levels of the ID would have changed the main findings of the current study. Our interest has been in determining whether ID-induced increases in visual feedback processing are related to changes in time-series structure [6,7,17]. The 2- and 5-bit ID levels were selected because feedforward-dominant processing is thought to operate under 2 bits with a transition to the engagement of closed-loop visual feedback processing by 5 bits: For example, under conditions of full visual feedback, Hoffman [14] estimated that below and above a critical-ID range of 3-to-4 bits, feedforward control is dominant and closed-loop visual feedback processing is engaged, respectively. In the current study, the 2- and 5-bit ID levels straddled that critical-ID range. Had intermediate ID levels been included in the visual feedback condition, a progressive reduction in *β* would have been expected. That expectation follows from our previous results where we found a systematic reduction of *β* over the ID levels of 2, 3, 4, and 5 bits [6, see Footnote 3 & Fig 3 bottom panel]. In the current no-visual feedback condition, strengthened pink noise was observed without a reliable difference between the ID-2 and ID-5 *β* values. We would not expect different *β* values at intermediate ID levels. Thus, adding more ID levels should result in a similar pattern of results as that of the current study: Namely, we would still expect a reliable ID by visual feedback interaction like that shown in Fig 7.

While including conditions where an increase in the ID was achieved through a reduction in target width, including variations in target display scale, and including additional ID levels would provide more detail about the influence of the ID and visual feedback conditions on movement amplitude time-series structure, doing so would come at a cost: Reliable time-series analyses require lengthy time series, which increase both the duration of experimental sessions and the likelihood of participant fatigue [31]. Consequently, there are constraints on the number of independent variables and levels of an independent variable that can be included in a study like the current one. Again, we do not think that our basic results would change if variations in ID had been realized through reductions in target width or if multiple ID or scale levels had been included. At the very least, the current study represents a reasonable initial attempt at addressing our hypotheses about the influence of the ID and visual feedback manipulations on movement amplitude time-series structure. Nevertheless, to gain additional detail, future research may consider experimental designs to accommodate the inclusion of the independent variables and the additional levels of the independent variables discussed here.

## Conclusion

The original motivation for this study was to test the possibility that the proximal, direct cause of increased movement amplitude time-series whitening with increases in ID was the ID-induced need to engage the available visual feedback through closed-loop processing. The current findings provided support for that hypothesis. Furthermore, the results suggest that *β* is uniquely sensitive to closed-loop visual feedback processing. Future research with the goal of quantifying the degree of closed-loop visual feedback engagement may benefit from assessments of motor-event time-series structure via *β*: Variations in *β* might predict the degree of closed-loop visual feedback processing in a similar fashion as variations in time-series structure predict neurological disease severity [32, Fig 3: Huntington’s disease; 33, Fig 5: Parkinson’s disease].

Last, the current study adds to other attempts to understand the processes and mechanisms underlying the structure of long sequences of discrete motor events [e.g., 32,34–36]. In particular, the current results are in accord with studies on a range of motor systems and tasks—for example, interval timing [37], manual aiming [38], precision grip-force production [39], and treadmill walking [40–44]—demonstrating time-series whitening, or decorrelation, when there was an internal-to-external shift in information processing [e.g., 35]. For example, when participants’ attention to an external task goal was amplified—by enriching movement-related feedback [39,43,44] or by inducing a negative-to-positive shift in mood [37]—time-series structure whitened. In addition, when time series from more than one motor-output variable were concurrently recorded, the time-series variable most relevant to the external task goal whitened, or decorrelated, relative to the less goal-relevant variable(s) [38–44]. In the current study, observations of a pink-to-white-noise shift in time-series structure can be understood as a shift from internal or feedforward information processing to external or feedback information processing. In that case, we believe the feedback processing is superimposed on existing feedforward processing [6,7,17]. The notion that superimposition of feedback processes on feedforward processes may account for shifts in time-series structure has been implied, at least, by other theory and modeling efforts [43,45,46].
